# Voice-Controlled Intelligent Personal Assistants in Health Care: International Delphi Study

**DOI:** 10.2196/25312

**Published:** 2021-04-09

**Authors:** Alena Ermolina, Victor Tiberius

**Affiliations:** 1 Faculty of Economics and Social Sciences University of Potsdam Potsdam Germany

**Keywords:** Delphi study, medical informatics, voice-controlled intelligent personal assistants, internet of things, smart devices

## Abstract

**Background:**

Voice-controlled intelligent personal assistants (VIPAs), such as Amazon Echo and Google Home, involve artificial intelligence–powered algorithms designed to simulate humans. Their hands-free interface and growing capabilities have a wide range of applications in health care, covering off-clinic education, health monitoring, and communication. However, conflicting factors, such as patient safety and privacy concerns, make it difficult to foresee the further development of VIPAs in health care.

**Objective:**

This study aimed to develop a plausible scenario for the further development of VIPAs in health care to support decision making regarding the procurement of VIPAs in health care organizations.

**Methods:**

We conducted a two-stage Delphi study with an internationally recruited panel consisting of voice assistant experts, medical professionals, and representatives of academia, governmental health authorities, and nonprofit health associations having expertise with voice technology. Twenty projections were formulated and evaluated by the panelists. Descriptive statistics were used to derive the desired scenario.

**Results:**

The panelists expect VIPAs to be able to provide solid medical advice based on patients’ personal health information and to have human-like conversations. However, in the short term, voice assistants might neither provide frustration-free user experience nor outperform or replace humans in health care. With a high level of consensus, the experts agreed with the potential of VIPAs to support elderly people and be widely used as anamnesis, informational, self-therapy, and communication tools by patients and health care professionals. Although users’ and governments’ privacy concerns are not expected to decrease in the near future, the panelists believe that strict regulations capable of preventing VIPAs from providing medical help services will not be imposed.

**Conclusions:**

According to the surveyed experts, VIPAs will show notable technological development and gain more user trust in the near future, resulting in widespread application in health care. However, voice assistants are expected to solely support health care professionals in their daily operations and will not be able to outperform or replace medical staff.

## Introduction

### Overview

With their expanding capabilities, voice-controlled intelligent personal assistants (VIPAs), such as Amazon Echo and Google Home, profoundly change the way people interact with technology [1]. Using natural language processing (NLP) or natural language understanding (NLU), as well as cloud data storage, VIPAs can process nearly any human request in real time, mimicking natural human communication [2]. Following the recent *Dr Google* phenomenon [3], conversational agents have quickly become a source of knowledge for health-related requests, providing information on various physical and mental health conditions [4]. Over the past years, advances in machine learning, particularly in neural networks, have enabled voice-based technologies to assist health professionals during medical consultation [5], provide diagnostic support [6], assist elderly people in their daily routines [7,8], and promote an overall healthier lifestyle [9]. Furthermore, intelligent assistants show potential in recognizing emotions and developing robot-human relationships with users [10,11], which can open a wide range of new applications for mental health patients, after-diagnosis support, and treatment of chronic conditions [12]. In particular, the current COVID-19 pandemic might trigger telemedical approaches involving VIPAs [13].

However, the potential to improve treatment efficiency and save costs comes with possible safety and privacy risks [5,14]. Incomplete or incorrect health-related information, such as first-aid instructions or medication recommendations, may lead to patient harm, especially when users blindly follow the instructions of VIPAs without understanding the limitations of the technology [15]. Serious privacy concerns about the use, disclosure, and protection of personal health data may also keep users from sharing health information and therefore have a large impact on customer acceptance of online health care applications [16].

Such conflicting influences on the adoption of VIPAs make it difficult to foresee their further development. At the same time, the disruptive potential of the technology in health care urges companies to innovate to be able to meet consumer requirements in the future [17]. The necessity to respond to market changes creates a need for an insightful technological forecast, which could support decision making within organizations, facilitate smooth innovation processes, and help companies to maintain or even advance their competitiveness [18]. Despite a wide selection of literature on intelligent voice assistants and their use in health care, the majority of studies have focused on specific therapy areas or functions, such as health coaching [19,20], disease detection [21], applications in psychotherapy [22,23], and others. The overall state and current progress of the field were recently examined by Laranjo et al and Montenegro et al [5,24]. However, studies using widely recognized foresight or forecasting methods, such as a Delphi study, that can shed light on the further development of conversational agents in health care do not exist to date. This paper aimed to fill this gap and present a comprehensive future scenario of the adoption of VIPAs in health care, based on a two-round expert online survey. In particular, the main technology trends, customer acceptance development, promising use cases, and possible regulation changes in the next 5 years have been examined.

With our findings, we contribute to ubiquitous health-related computing services (uHealth) and more specifically VIPAs by forecasting their further development in regard to technology, consumer acceptance, potential use cases, and privacy and data protection regulations, and by drawing practical conclusions for health care providers.

The remainder of the paper is organized as follows. The next section provides a brief technical background of VIPAs and covers the main factors affecting adoption in health care. The methodology section provides details on the Delphi technique and the main steps carried out during the study. Conclusively, the research findings are presented, followed by a discussion of research limitations and suggestions for future research.

### Background

A voice assistant is an artificial intelligence–powered computer system that aims to imitate human intelligence while engaging in realistic conversations with users [25]. The current most popular examples are Amazon Alexa, Google Assistant, Apple Siri, Microsoft Cortana, and Samsung Bibxy [26]. As of 2020, VIPAs are integrated in numerous devices, like smartphones, speakers, smartwatches, smart televisions, cars, headphones, game consoles, and household appliances [26]. Unless the system is deactivated by the user, the software always listens for trigger keywords, such as “Alexa” and “Hey Siri,” and automatically starts audio recording when awakened [2]. The request is then transmitted to the cloud, processed using NLU, and assigned to a specific intention. Depending on the inquiry, the server will provide relevant information for the voice assistant to be presented to the user or execute tasks with numerous voice applications and connected devices [2].

Unlike previous intelligent systems, VIPAs can respond to much larger numbers of requests owing to the constant internet connection and access to rapidly growing amounts of services developed directly by VIPA providers or external companies like Uber, McDonalds, and Disney [2]. Because voice built-in capabilities (eg, “Alexa Skills” and “Google Actions”) are stored in the cloud, there is no need for users to download or install them in contrast to smartphone apps. Third-party voice extensions can be published for the general public in a voice app store like *Alexa Skills Store* or used exclusively within an organization, which provides a great opportunity for companies to use VIPAs for their specific needs [27].

Petrock highlights that 33.8% of people from the total US population are currently using a voice assistant at least monthly for various requests [26]. This number is predicted to increase to 36.6% in 2021 [26]. Constantly improving, VIPAs can execute over 100 different tasks, covering the ability to control home appliances, answer numerous questions, set reminders for medications or tasks, shop online, and others [7,28]. In the health care field, various research trials using virtual assistants have shown major advancements in physical training, diet adjustments, accessibility to health information, etc [5,29,30]. However, according to the number of applications already available on the market, the use of voice assistants in the health care context is in its early days in comparison to entertainment, information search, navigation, and calling [26]. Several innovative health care providers, like Mayo Clinic, Boston Children’s Hospital, Atrium Health, and Deloitte, have introduced their voice solutions for Alexa ranging from first aid help and home care treatment to medical appointment scheduling and patient communication [31]. Still, most health care companies are hesitant to adopt VIPAs, primarily due to legal compliance and privacy concerns. At present, given that private health care data may be shared by a patient using a virtual assistant, health care providers have the option of getting access to Amazon’s Health Insurance Portability and Accountability Act–compliant solution for US-based Alexa Skills or building their own voice assistant [31].

Another important barrier for the adoption of VIPAs in health care is privacy concerns. Amazon, Apple, Google, Facebook, and Microsoft have all been using third-party human contractors who listen to user audio recordings to improve the quality of NLU [31]. Since private consumer data had been shared without user consensus, major data privacy concerns can arise from the user side.

## Methods

### Delphi Study

To generate a plausible scenario for the use of VIPAs in health care, a two-stage Delphi study was conducted online. The Delphi method is a forecasting technique that relies on experts in a particular field to identify technology developments and trends [32]. According to research guidelines, experts anonymously provide their answers to a standardized questionnaire over at least two rounds [33]. The interim results of each round are summarized and fed back to the experts during the next stage to narrow the statistical spread and facilitate concurrence among the participants [34-38].

As Wright et al described, a Delphi study is especially suitable in cases requiring human judgmental input owing to missing historical or technical data, such as forecasting the development of emerging technologies [39]. In contrast to causal-deterministic natural development processes, such as the weather, societal changes are based on human intentions, social interactions, and coincidence [40]. Therefore, societal forecasts can be deduced from subjective expert knowledge and experience-based assessments [41]. The Delphi method surpasses similar interactive group techniques regarding accuracy and efficacy [42-44], and has been frequently used in various fields. In their bibliometric analysis, Flostrand et al found 175 Delphi-related papers in the business field and 1462 papers in the health care field in the period between 1975 and 2017 [45]. Although health care research often aims to find a consensus in general, without a future focus, it has also been applied to examine future developments and tendencies [46-49]. Therefore, the Delphi technique is appropriate to support health care providers’ strategic decision making in regard to VIPA implementation.

### Formulation of Projections

The topic under investigation is the future of VIPAs in health care within the next 5 years. This timeframe was used owing to the rapid development of the technology, which complicates forecasting the further development regarding VIPAs within a wider time span. Furthermore, the given time period serves the purpose of this study to provide health care organizations with clear guidance regarding the necessity and application of VIPAs in the near future.

According to our knowledge, the future of VIPAs in health care has not been examined in other studies yet. Hence, an exploratory approach was applied to achieve a broad rather than deep scenario. We determined four thematic sections (technology, consumer acceptance, potential use cases, and privacy and data protection regulations) that were supposed to be investigated. As a consequence of this multisection approach, we omitted deeper investigations of each topic in greater detail to ensure an appropriate length of the questionnaire and thus to avoid a low response and high dropout rate from the experts. We considered 20 projections as appropriate for the questionnaire. An equal number of projections per section was not considered necessary.

The formulation of the projections followed a systematic process. First, we conducted desk research (based on academic journals; publications by relevant health associations, consultancies, and state authorities; news articles; and social media posts) and brainstormed to identify relevant issues for the four sections. Second, a small expert panel consisting of two information technology (IT) and two health care experts reviewed the list of issues, without adding or removing items. Third, the issues were transformed into projections. We aimed to formulate the projections in a plausible way such that they could be agreed to. However, implausible statements could also be incorporated into a Delphi study, since both the acceptance of a plausible projection and the rejection of an implausible projection represent the same general trend and can equally contribute to the resulting scenario [50]. Fourth, the list of projections was forwarded to the small expert panel again for review. As a consequence, some formulations became more precise and concise. The final list of projections is presented in Textbox 1.

**Delphi projections (within the next 5 years).**

**Cluster 1: Technology**

1. Voice-controlled intelligent personal assistants (VIPAs) will be able to give solid medical advice based on personal medical information, instructions, and plans.

2. VIPAs will be able to hold a human-like conversation.

3. VIPAs will be able to take into account patients’ emotions, moods, and traits.

4. VIPAs will outperform humans in the quality of anamnesis and the ability to have the complete medical history available.

**Cluster 2: Consumer Acceptance**

5. From a patient’s perspective, VIPAs will provide a frustration-free user experience.

6. A high number of patients will regularly use VIPAs for health-related inquiries.

7. Owing to simple use, a high share of elderly people will use VIPAs.

8. The majority of patients will prefer human communication over VIPA communication when in need of psychological support.

9. The majority of patients will prefer VIPAs owing to time and cost savings.

10. Patients will consider trust in content providers (owners of voice applications, such as hospitals and pharmaceutical companies) as more important than trust in VIPA providers (eg, Amazon and Google).

**Cluster 3: Potential Use Cases**

11. VIPAs will be widely used as remote and in-house real-time anamnesis tools.

12. VIPAs will be widely used as diagnostic support tools for diseases that can be detected through speech characteristics.

13. VIPAs will be widely used as hands-free instruction tools for medical staff (eg, in sterile environments).

14. VIPAs will be widely used as communication tools between medical staff and patients.

15. VIPAs will be widely used as self-therapy tools for patients outside the clinic.

**Cluster 4: Privacy and Data Protection Regulations**

16. The majority of VIPAs will be compliant with the applicable regulations regarding protected health information.

17. The majority of VIPA providers will discontinue the use of subcontractors to increase trust in data security.

18. Government privacy concerns will greatly decrease.

19. The majority of customers will deliberately share sensitive health information with VIPAs.

20. Too strict regulations will inhibit VIPAs to provide reliable high-quality medical help services.

### Panelists

Expert selection is essential for the quality of the resulting forecast [36,51-54]. Following a purposive sampling approach [55], the experts were chosen based on their expertise in the field [33,54,56]. For this study, we searched for the following expert groups: (1) managers from VIPA manufacturers or developers (with job titles such as business development manager and product manager), (2) IT experts from VIPA manufacturers and developers (with job titles such as data scientist, software development engineer, and UX designer), (3) managers from hospitals (with job titles such as executive director, chief innovation officer, and chief technology officer), (4) physicians from hospitals, (5) researchers (especially professors) working in the academic fields of health care management, health care economics, or health care law, (6) representatives of state authorities related to health care and nongovernmental health care associations, and (7) health care consultants. Eligible participants were identified through scholarly publications, conference presentations, and field-specific expert awards related to VIPAs, and job descriptions or posts on the professional social network *LinkedIn*.

Overall, 154 experts were invited to complete the survey. Of these, 35 experts participated in the first round and 27 remained in the second round. The dropout rate of 22.9% is below the 30% rate that is considered acceptable [57,58]. The majority of Delphi panels involve 15 to 35 participants [57], acknowledging seven [59] to 10 [58] as the minimum. Especially in regard to the infancy of the field, the panel selection in this study is considered adequate to achieve the research goal. The panel structure is presented in Table 1. Unfortunately, we were unable to recruit participants from state authorities related to health care or from nongovernmental health care associations, as well as health care consultants. The low proportion of female participants represents the gender distribution in the field of artificial intelligence. Similarly, the prevalence of experts from the North, Central, and South America (AMER) region mirrors the relatively high level of voice technology development and VIPA application in health care in the United States.

**Table 1:** Panel demographics.

Characteristic			First round (n=35), n (%)		Second round (n=27), n (%)
**Gender**					
	Male	25 (71)		17 (63)	
	Female	9 (26)		9 (33)	
	Other	1 (3)		1 (4)	
**Age (years)**					
	<30	3 (9)		1 (4)	
	30-40	10 (29)		9 (33)	
	41-50	9 (26)		7 (26)	
	51-60	10 (29)		8 (30)	
	>60	3 (9)		2 (7)	
**Affiliation**					
	Information technology expert (VIPA^a^ firm)	5 (14)		4 (15)	
	Manager (VIPA firm)	9 (26)		8 (30)	
	Manager (hospital)	8 (23)		5 (19)	
	Physician (hospital)	7 (20)		3 (11)	
	Researcher (academia)	6 (17)		7 (26)	
	Representative (authorities or association)	0 (0)		0 (0)	
	Health care consultant	0 (0)		0 (0)	
**Region**					
	AMER^b^	17 (49)		17 (67)	
	EMEA^c^	14 (40)		9 (30)	
	APAC^d^	4 (11)		1 (4)	

^a^VIPA: voice-controlled intelligent personal assistant.

^b^AMER: North, Central, and South America.

^c^EMEA: Europe, Middle-East, and Africa.

^d^APAC: Asia Pacific.

### Data Collection

The questionnaire covered the 20 projections presented in Textbox 1. The experts were asked to provide their level of agreement with the future statements, using a 4-point Likert scale (“do not agree,” “somewhat disagree,” “somewhat agree,” and “agree”). An even-numbered Likert scale was used to avoid neutral responses [41]. Further, demographic data, such as gender, age, profession, and location, were collected from the participants (Table 1).

To minimize the possibility of misinterpretation of the statements and prevent technical problems with the survey tool, pretests were conducted prior to each round. The first survey round was executed between September 18 and 29, 2020, followed by the second round between October 7 and 14, 2020.

## Results

### Descriptive Statistics

According to Delphi study guidelines, the most probable future scenario is generated from the accumulation of the group consensus on each projection. The panel’s agreement on each statement is concluded from the aggregation of individual assessments. The following numerical values were assigned to the response options to enable the calculation of statistical distribution: “do not agree,” 1; “somewhat disagree,” 2; “somewhat agree,” 3; and “agree,” 4.

Considering resistance toward outliers and the potential risk of statistical biases, the median is favored over the mean as the statistical average for Delphi studies [36,41]. Scattering of the responses is evaluated by IQR, which is the difference between the upper quartile (x_0.75_) and the lower quartile (x_0.25_). A low IQR indicates a high level of agreement, whereas a high IQR reflects a high level of discord [41].

Table 2 depicts the detailed results for both rounds. In the second round, no projection received strong agreement (median 4). The experts somewhat agreed (median 3) with most of the projections, namely projections 1, 2, 6, 7, 8, 10, 11, 13, 14, 15, 16, and 19. They somewhat disagreed with projections 3, 4, 5, 9, 12, 17, and 20, and even strongly disagreed with projection 18.

During the second round, only slight changes occurred. The median did not change for the majority of the projections. For projections 3 and 5, the experts revised their assessment from partial agreement to partial disagreement. Further, the median changed from slight to strong disagreement for projection 18. The IQR did not rise for any projection except for projection 9, showing increased insecurity in the panel, but reduced (as aimed) for eight projections, namely projections 2, 4, 7, 10, 13, 16, 17, and 18. Generally, the IQR evaluation shows a high level of consensus among participants. Additionally, to measure the level of consensus, Kendall *W* coefficient of concordance was calculated for both rounds [60]. The value can vary from 0 to 1, but is somewhat difficult to interpret, as it does not depict a linear rise. However, closer proximity to 1 reflects stronger agreement [61,62]. Kendall *W* equaled 0.1188 (χ^2^_19_=78.9689, P<.001) for the first round and 0.2948 (χ^2^_19_=151.2496, P<.001) for the second round of the Delphi study. As expected, the level of consensus among panelists increased in the course of the study. In light of the level of consensus and only minor variations between the rounds, no further iterations were required.

**Table 2:** Descriptive statistics.

Cluster and projection		First round (n=35)				Second round (n=27)				Difference			
		X_0.25_^a^	X_0.5_^b^	X_0.75_^c^	IQR	X_0.25_	X_0.5_	X_0.75_	IQR	X_0.25_	X_0.5_	X_0.75_	IQR
**Cluster 1: Technology**													
	1	2	3	3	1	2	3	3	1	0	0	0	0
	2	2	3	4	2	2	3	3	1	0	0	-1	-1
	3	2	3	3	1	2	2	3	1	0	-1	0	0
	4	1	2	3	2	1	2	2	1	0	0	-1	-1
**Cluster 2: Consumer acceptance**													
	5	2	3	3	1	2	2	3	0	-1	0	0	0
	6	3	3	4	1	3	3	4	1	0	0	0	0
	7	2	3	4	2	2	3	3	1	0	0	-1	-1
	8	2	3	4	2	2	3	4	2	0	0	0	0
	9	2	2	3	1	1	2	3	2	-1	0	0	1
	10	2	3	4	2	3	3	4	1	1	0	0	-1
**Cluster 3: Potential use cases**													
	11	2	3	3	1	2	3	3	1	0	0	0	0
	12	2	2	3	1	2	2	3	1	0	0	0	0
	13	2	3	4	2	2	3	3	1	0	0	-1	-1
	14	2	3	3	1	2	3	3	1	0	0	0	0
	15	2	3	3	1	2	3	3	1	0	0	0	0
**Cluster 4: Privacy and data protection regulations**													
	16	2	3	4	2	3	3	3	0	1	0	-1	-2
	17	2	2	3	1	2	2	2	0	0	0	-1	-1
	18	1	2	3	2	1	1	2	1	0	-1	-1	-1
	19	2	3	3	1	2	3	3	1	0	0	0	0
	20	2	2	3	1	2	2	3	1	0	0	0	0

^a^X_0.25_: lower quartile.

^b^X_0.5_: median quartile.

^c^X_0.75_: upper quartile.

### Scenario

According to the surveyed experts, VIPAs will show notable technological development and gain more trust among users in the next 5 years, resulting in their widespread utilization in health care. However, voice assistants are expected to only support health care professionals in their daily operations and will not be able to outperform or replace medical staff.

Within the next 5 years, the respondents expect a high percentage of patients to use VIPAs for health-related inquiries on a regular basis. Remarkably, a large proportion of elderly people will use voice assistants owing to their screen-free simple interface. Although experts agreed on the ability of VIPAs to hold a human-like conversation in the next 5 years, the given timeframe is still assessed as too short for the technology to be able to provide a frustration-free user experience.

Anticipating extensive use of voice assistants in health care, experts mentioned communicational, anamnesis, and self-therapy tools for patients and medical staff as the most promising use cases. Still, questioning the ability of VIPAs to recognize and take patients’ emotions, moods, and traits into consideration, wide application of conversational assistants as diagnostic tools is not expected by 2025.

Pursuant to the survey outcome, the majority of voice assistants will be compliant with the applicable health information regulations within the time span of 5 years. Furthermore, data compliance together with trust in content providers, such as hospitals and pharmaceutical companies, might result in higher user willingness to share sensitive health information with VIPAs. While experts strongly rejected the possibility of a decrease in governmental privacy concerns, strict regulations capable of preventing VIPAs from providing medical help services are not expected.

## Discussion

### Discussion of the Results

In the technology section, the experts expressed discordant opinions about three of four projections. While the majority of responders agreed with the ability of VIPAs to provide solid medical advice based on patients’ medical information, instructions, and plans within the next 5 years, 48% of the panelists rejected the statement. Hence, no clear group opinion could be derived for projection 1. Since not only technological development but also a comprehensive legal framework is required to enable voice assistants to provide medical help, some participants might reject the projection taking into account broad legal and privacy concerns. The presence of expert preoccupation with legal issues was confirmed in the fourth cluster of this study.

Likewise, projection 2 was accepted by the majority (52%) of panelists. Still, 48% of panelists expressed doubt about the potential of VIPAs to have a human-like conversation within the next 5 years. The group skepticism regarding the ability of VIPAs to accurately imitate human skills is reinforced in projections 3, 4, 5, 8, and 9. In detail, 81% of the interviewees believed voice assistants will not be able to outperform humans in the quality of anamnesis in the next 5 years. Projection 5, which stated that VIPAs can provide a frustration-free user experience, was also rejected. Furthermore, 66% of the panel expected patients to prefer humans over voice assistants when in need of psychological support.

Questioning the likelihood of the interchangeability between voice assistants and medical staff in the near future, the experts agreed with the widespread use of VIPAs as support tools. With a high level of consensus, 85% of the panel members anticipated a high number of patients to regularly use VIPAs for health-related inquires. Notably, no expert strongly disagreed with the projection.

Examining the most promising use cases, the panelists agreed on the application of VIPAs by medical staff (eg, in sterile environments, with a majority of 74%). Accordingly, 70% of the group expected extensive use of conversational agents among elderly people and 73% believed patients and health care professionals will use voice assistants for communication. Projection 15 suggesting the use of voice assistants as self-therapy tools was accepted by 59% of the experts. The fact that 41% of the responders expressed doubt about the given use case might reflect the general trend of expert skepticism toward the employment of voice assistants without the presence of health professionals.

The assumption of a potential decrease in the government’s privacy concerns was strongly rejected. Only one expert somewhat agreed with the statement, with the rest of the group showing slight or strong disagreement. Furthermore, 85% of the panelists declared they do not expect VIPA providers to stop using subcontractors who listen to user audio files to improve the quality of NLU. Thus, data concerns might remain in place, requiring a new set of regulations. Further research is required to obtain a better understanding of the available tools and the amount of time it would take to address regulatory issues.

From the scenario, several practical implications for health care providers can be concluded, which have been summarized in Textbox 2.

**Practical implications for health care providers (within the next 5 years).**

- Voice-controlled intelligent personal assistant (VIPA) technology will be mature.
- VIPAs will be accepted by patients, including elderly people.
- VIPAs will be used for regular anamneses, medical staff support, staff-patient communication, and self-therapy.
- Privacy and data protection issues will not harm the dissemination of VIPAs.

### Limitations and Future Research

As this study involves early research on the future use of VIPAs in health care, several limitations apply to this study, which could also provide guidance for future research. First, the Delphi technique develops the most probable future scenario based on experts’ present knowledge. Thus, although the method outperforms comparable interactive group forecasting techniques [42-44], the Delphi study cannot guarantee the exact realization of the forecast. Second, the range of addressed questions was limited to keep a sufficient length of the survey and avoid low response rates from the experts. Therefore, only existing features and use cases, which are currently in the early days of their implementation, were examined. The probability of the emergence of new technologies and applications for VIPAs was not investigated.

This study aimed to provide clear guidance for health care companies regarding the current necessity to focus their efforts on voice technology. Thus, a relatively short time frame of 5 years was chosen. Especially for the Europe, Middle-East, and Africa (EMEA) and Asia Pacific (APAC) regions, where the use of voice assistants in health care is in its infancy, the given time span could potentially interfere with the experts’ future assumptions.

Currently, the use of voice assistants in health care varies widely across countries. The quality of NLP/NLU in English greatly outperforms other languages. Further factors, including the total user base, tools available for third-party developers, data protection regulations, and cultural differences, determine the adoption of VIPAs in general and in particular in health care. During this study, no large differences were detected comparing AMER, EMEA, and APAC expert assessments. Still, a study covering a specific region might provide more insightful forecasts of VIPA development in a particular geographical area.

Despite the widespread use of the Delphi technique, a number of claims have been made regarding its methodology. First, anonymity is one of the central characteristics of the Delphi technique, which aims to encourage experts to provide true and not socially likeable assessments. However, Sackman points out that anonymity can lead to hasty judgments as a result of experts’ assurance that there is no necessity to defend their responses [63]. Second, the Delphi technique requires disclosure of the interim results of each round to generate a group opinion that can be claimed to be representative [64]. Some scholars argue that independent judgement is violated once the panelists know how others have evaluated each item [64]. Further, this disclosure can encourage outliers to revise their assessments owing to group pressure and not because of a changed opinion. Hence, a reduced IQR rate in Delphi studies can correspond to the problem of group thinking rather than higher consensus among experts [36,64,65].

### Conclusion

We conducted an international Delphi study and derived a plausible scenario of the future of voice assistants in health care within the next 5 years. Twenty projections were designed and evaluated by an internationally recruited panel consisting of voice experts, as well as medical professionals and representatives of academia, health authorities, and nongovernmental health associations having broad experience with voice technology.

With a high level of consensus, the experts anticipate widespread application of VIPAs in various health care domains in the next 5 years. Although conversational assistants are not expected to replace medical workers, their use as operational supporting tools for health care professionals has strong potential in the industry. In detail, the panelists agreed with the capability of VIPAs to support elderly people and to be widely used as anamnesis, informational, self-therapy, and communicational tools by patients and health care professionals. Although users’ and governments’ privacy concerns are not expected to decrease in the near future, the panel members believe that strict regulations capable of preventing VIPAs from providing medical help services will not be imposed. To be able to meet consumer expectations and withstand competition within the next 5 years, health care companies are advised to carefully observe current research and development activities in the field of conversational artificial intelligence and allocate resources to optimize business processes using VIPAs.

This was an exploratory study on the future of voice assistants in health care. Hence, a broad rather than deep scenario approach was applied. Further studies might take a deeper look at one of the four clusters examined in this study or focus on a specific geographical area to provide more detailed insights.

## Abbreviations

AMER: North, Central, and South America
APAC: Asia Pacific
EMEA: Europe, Middle-East, and Africa
IT: information technology
NLP: natural language processing
NLU: natural language understanding
VIPA: voice-controlled intelligent personal assistant
